# Determinants of Hospital Length of Stay Among Trauma Patients in Sudan: A Retrospective Study (2009–2014)

**DOI:** 10.7759/cureus.104185

**Published:** 2026-02-24

**Authors:** Abdelrahim Shaaeldin, Mohsen N Alhajuj, Mohamed N Alhajuj, Sara M Altom, Faris M Elmahdi

**Affiliations:** 1 Orthopedics and Traumatology, Al Rayan National College of Medicine, Madinah, SAU; 2 Medicine and Surgery, Al Rayan National College of Medicine, Madinah, SAU; 3 Basic Medical Sciences, Al Rayan National College of Medicine, Madinah, SAU

**Keywords:** determinants, emergency medicine, hospital length of stay, predictors, trauma patients

## Abstract

Background: Hospital length of stay (LOS) is a critical indicator of healthcare efficiency and resource utilization. Understanding the factors influencing LOS among trauma patients can help improve hospital management, reduce costs, and enhance patient outcomes.

Objective: This study aimed to assess LOS and identify key determinants influencing prolonged hospitalization at El-Obeid Teaching Hospital, Sudan.

Methods: This retrospective observational study was conducted among trauma patients at El-Obeid Teaching Hospital in Sudan. All data of trauma patients admitted between 2009 and 2014 were collected. A structured data collection tool was used to collect data from patient charts that include demographic characteristics (i.e., age, gender) and clinical characteristics such as diagnosis, admission source, mechanism of trauma, type of intervention, and duration of hospital stay. All statistical data were analyzed using Statistical Package for the Social Sciences (SPSS) version 26 (IBM, SPSS Inc., USA).

Results: A total of 322 trauma patients were reviewed and analyzed. The patients' mean age was 31.1 years. Male patients were prevalent (74.8%). Admission due to an accident was common (84.2%), and fracture was the most commonly diagnosed type of trauma (44.4%). The prevalence of patients with prolonged LOS (≥7 days) was 47.8%. Results suggest that increasing age, patients with fractures, hospital admission due to road traffic accidents (RTAs), and patients who underwent surgery were associated with increasing duration of hospital stay. In multivariate regression analysis, increasing age and surgical procedure were identified as the significant independent risk factors for prolonged LOS.

Conclusion: Nearly half of trauma patients had hospital admission for seven days or more. Younger patients who were admitted due to RTA and did not undergo surgical intervention were more likely to have a shorter LOS. Further larger studies are required to provide more insights about the hospital admission duration of trauma patients and the factors that influence it in our region.

## Introduction

A widely accepted and important indicator for evaluating the effectiveness of healthcare, resource use, and patient-care quality is hospital length of stay (LOS) [1]. A longer LOS is frequently suggestive of increased injury severity, the emergence of comorbidities, or complicated recovery processes in the setting of trauma, where injuries can be abrupt, severe, and diverse [2]. Therefore, comprehending and maximizing LOS is not only an administrative issue; it is also essential to enhancing therapeutic results and expediting the provision of emergency and surgical care. In the end, cutting down on needless hospital stays may help the patient and the healthcare system by lowering healthcare expenses dramatically, freeing up scarce bed capacity, and lowering the risk of hospital-acquired infections [3].

Numerous demographic, clinical, and organizational factors are among the many complicated and multidimensional factors that contribute to LOS in trauma patients. Due to comorbidities, decreased physiological reserve, and an increased risk of post-injury sequelae, the research currently in publication consistently finds that advanced age is a strong predictor of extended hospitalization [4,5]. Additionally, because of the need for operative management, postoperative monitoring, and rehabilitation, the type and severity of injury, especially fractures and traumatic brain injuries, as well as the need for surgical intervention, are strongly associated with longer inpatient stays [6,7]. Injury patterns and the ensuing healing paths are also influenced by the trauma mechanism, such as the difference between falls and traffic accidents (RTAs).

Restrictions in infrastructure, resources, and specialized care make treating trauma more difficult in low- and middle-income countries (LMICs) [8]. Hospitals where efficient hospital administration and planning depend on a thorough grasp of local LOS patterns and their causes. The lack of region-specific data on this topic, however, makes it difficult for doctors and healthcare managers to create focused plans for enhancing patient flow and treatment efficiency in these settings with limited resources.

In order to determine the length of hospitalization and the major factors causing extended hospitalization among trauma patients admitted to El-Obeid Teaching Hospital in Sudan, this study was conducted. The results of this study will offer important data to guide hospital policy, maximize resource allocation, and improve trauma care delivery catered to the unique requirements and difficulties of the area by clarifying the local determinants linked to prolonged LOS.

## Materials and methods

Study design and setting

This study employed a retrospective, observational, cross-sectional design. Data were collected from the medical records of trauma patients admitted to El-Obeid Teaching Hospital, Sudan, over a six-year period, from January 1, 2009, to December 31, 2014. El-Obeid Teaching Hospital is a major referral center in North Kordofan State, providing emergency and specialized surgical services to a large catchment population. The study was conducted after obtaining ethical approval from the institutional review board of the hospital.

Study population and sampling

The study population included all patients admitted with a primary diagnosis of trauma during the study period. A census sampling approach was adopted, whereby all eligible trauma patients admitted during the specified time frame were included. Accordingly, an a priori sample size or power calculation was not applicable.

Inclusion criteria comprised patients of all ages and both genders who were admitted with a trauma diagnosis and had sufficiently complete medical records. Patients who were dead on arrival or had missing key variables, including LOS or mechanism of injury, were excluded. From the initial pool of records, 322 patient charts met the inclusion criteria and were reviewed. However, complete LOS data were available for 247 patients, who constituted the final sample for the primary outcome analysis.

Data collection instruments and procedures

Data were extracted using a structured, pre-tested data collection form specifically designed for this study. The form captured demographic variables (age and gender) and clinical characteristics, including diagnosis category, admission source, mechanism of trauma, and type of intervention (surgical or non-surgical management).

The primary outcome variable was LOS, calculated as the number of days from hospital admission to discharge. LOS was categorized as prolonged if the duration was seven days or more. To ensure consistency and data quality, the data collection tool underwent pilot testing, and data collectors received standardized training on variable definitions and extraction procedures to minimize inter-observer variability.

Data management and statistical analysis

Collected data were cleaned, coded, and entered into Statistical Package for the Social Sciences (SPSS) Statistics version 26.0 (IBM, SPSS Inc., USA) for analysis. Descriptive statistics were used to summarize demographic and clinical characteristics. Continuous variables were presented as means with standard deviations or medians with ranges, as appropriate, while categorical variables were presented as frequencies and percentages.

Bivariate analysis using the chi-square test was performed to assess associations between independent variables and prolonged LOS. Variables that demonstrated statistical significance in bivariate analysis were entered into a multivariate logistic regression model using the enter method to identify independent predictors of prolonged LOS. Results were reported as adjusted odds ratios (AORs) with 95% confidence intervals (CIs). A p-value of less than 0.05 was considered statistically significant. Extreme LOS values were retained to reflect real-world clinical practice.

Ethical considerations

Ethical approval for this retrospective study was obtained from the Ethical Review Committee of El-Obeid Teaching Hospital (Approval No.: 20-A-1). As the study involved analysis of existing medical records without direct patient contact, the requirement for informed consent was waived. Patient confidentiality was maintained throughout the study by anonymizing all data during extraction and analysis. Personal identifiers were removed, and each record was assigned a unique study code. All data were stored on password-protected computers accessible only to the research team.

## Results

This study analyzed 322 trauma patients. As described in Table 1, the patients' mean age was 31.1 (SD 21.2) years, with 59% aged 30 years or younger. Male patients (74.8%) were predominantly higher than female patients (25.2%). The most commonly diagnosed injury was fracture (44.4%), while the accident was the most prominent admission source (84.2%). RTA was the most common mechanism of trauma (29.5%). Most of the patients did not undergo surgical intervention (71.7%). The median days of hospital stay were 6.00 days (0-300), and 47.8% had a prolonged duration of hospital stay (≥7 days) (Table 1). 

**Table 1:** Demographic and clinical characteristics of the patients (n=322) RTA: Road traffic accident; LOS: Length of stay

Study Variables	N (%)
Age in years (mean ± SD)	31.1 ± 21.2
≤30 years	190 (59.0%)
>30 years	132 (41.0%)
Gender	
Male	241 (74.8%)
Female	81 (25.2%)
Diagnosis	
Fracture	143 (44.4%)
Head and neck injury	19 (05.9%)
Soft tissue injury	21 (06.5%)
Abdominal trauma	50 (15.5%)
Others	89 (27.6%)
Admission source	
Accidents	271 (84.2%)
Clinic	46 (14.3%)
Health insurance	03 (0.90%)
Referred	02 (0.60%)
Mechanism of trauma	
RTA	95 (29.5%)
Fall	89 (27.6%)
Assault	42 (13.0%)
Others	96 (29.8%)
Type of intervention	
Surgery	91 (28.3%)
Non-surgery	231 (71.7%)
Length of hospital stay in days, median (min-max) (n=247)	6.00 (0 – 300)
Prolonged LOS (≥7 days)	118 (47.8%)
Short LOS (<7 days)	129 (52.2%)

Examining the relationship between the LOS among the demographic and clinical characteristics of the patients found that the older age group (X^2^=7.062; p=0.008), patients who had fracture (X^2^=6.559; p=0.010), those involved in RTA (X^2^=14.202; p=0.003) and those who had surgical intervention (X^2^=3.945; p=0.047) were more likely to have prolonged duration of hospital stay (Table 2).

**Table 2:** Multivariate logistic regression analysis to determine the significant independent predictors of longer duration of hospital stay (n=247) AOR: Adjusted odds ratio; CI: Confidence interval; RTA: Road traffic accident **Significant at p<0.05 level.

Factor	AOR	95% CI	P-value
Age group			
≤30 years	Ref		
>30 years	2.345	1.350 – 4.073	0.002**
Diagnosis			
Non-fracture	Ref		
Fracture	1.385	0.781 – 2.455	0.265
Mechanism of trauma			
RTA	0.294	0.117 – 0.738	0.009**
Fall	1.586	0.774 – 3.247	0.207
Assault	1.209	0.605 – 2.415	0.592
Others	Ref		
Type of intervention			
Surgery	1.966	1.095 – 3.530	0.024**
Non-surgery	Ref		

A multivariate logistics regression analysis was subsequently performed (Table 3) to determine the significant independent predictors of prolonged duration of hospital stay. The results revealed that compared to the younger age group, the older age group had an increased risk of prolonged LOS by at least 2.35 times higher (AOR=2.345; 95% CI=1.350 - 4.073; p=0.002). Compared to non-surgical patients, patients who underwent surgery had an increased risk of prolonged LOS by at least 1.9 times higher (AOR=1.966; 95% CI=1.095 - 3.530; p=0.024). However, compared to other trauma mechanisms, patients who were admitted to the hospital due to RTA were at lower risk of prolonged LOS with decreased odds of at least 80% (AOR=0.294; 95% CI=0.117 - 0.738; p=0.009). No significant effects were observed between the prolonged duration of hospital stay in relation to diagnosis after adjustment to a regression model (p=0.265) (Table 3).

**Table 3:** Relationship between length of hospital stay and demographic and clinical characteristics of patients (n=247) RTA: Road traffic accident ^§^P: p-value has been calculated using the chi-square test. **Significant at p<0.05 level.

Factor	Length of Hospital Stay		X^2^	P-value^§^
	Prolonged N (%) (n=118)	Short N (%) (n=129)		
Age group				
≤30 years	59 (50.0%)	86 (66.7%)	7.062	0.008**
>30 years	59 (50.0%)	43 (33.3%)		
Gender				
Male	91 (77.1%)	96 (74.4%)	0.244	0.621
Female	27 (22.9%)	33 (25.6%)		
Diagnosis				
Non-fracture	54 (45.8%)	80 (62.0%)	6.559	0.010**
Fracture	64 (54.2%)	49 (38.0%)		
Admission source				
Non-accident	16 (13.6%)	24 (18.6%)	1.156	0.282
Accident	102 (86.4%)	105 (81.4%)		
Mechanism of trauma				
RTA	39 (33.1%)	38 (29.5%)	14.202	0.003**
Fall	28 (23.7%)	34 (26.4%)		
Assault	25 (21.2%)	09 (07.0%)		
Others	26 (22.0%)	48 (37.2%)		
Type of intervention				
Surgery	43 (36.4%)	32 (24.8%)	3.945	0.047**
Non-surgery	75 (63.6%)	97 (75.2%)		

## Discussion

This study investigated the LOS and its determinants among trauma patients visiting El-Obeid Teaching Hospital in Sudan. The findings contribute meaningfully to the existing literature, particularly given the scarcity of data from developing countries on hospital LOS among trauma patients. Such research is crucial for enhancing healthcare delivery, optimizing resource utilization, reducing costs, improving patient outcomes, and informing policy and clinical practices. The findings should be interpreted within the context of the study period (2009-2014) and may not fully reflect current trauma care practices; however, they provide valuable insight into trauma care patterns in a resource-limited setting.

The results showed a median hospital LOS of six days (range: 0-300 days), with 47.8% experiencing prolonged stays (≥7 days). These findings are comparable to previously published trauma studies reporting similar median hospitalization durations [9]. However, other studies have demonstrated variability in the prevalence of prolonged LOS across different populations and healthcare systems [10,11]. These differences may be attributed to variations in definitions of prolonged stay, injury severity profiles, and healthcare infrastructure. Early assessment and timely intervention upon admission may contribute to reducing LOS through prompt injury management and complication prevention.

Multiple studies have documented that advanced age is associated with a longer LOS, which is consistent with our findings [12]. Older patients had over twice the odds of prolonged hospitalization compared to younger ones (AOR=2.345; p=0.002). This association may be attributed to increased injury complexity, pre-existing comorbidities, reduced physiological reserve, slower healing processes, and higher rates of complications among older individuals. However, a study by Alnahari and A’aqoulah reported that older patients were less likely to remain in the emergency department compared to younger patients, which may be explained by differences in trauma patterns, clinical settings, and study design [13].

Our findings showed no significant association between gender and LOS, differing from results reported by Alharbi et al. and Kashkooe et al., where male gender was linked with longer hospital stays [9,11]. Conversely, other studies have reported variability in LOS according to gender and demographic characteristics [12]. These inconsistencies may reflect demographic, cultural, and methodological differences. Additionally, Roshanaei et al. found that gender, trauma mechanism, and hospitalization history influenced LOS during the COVID-19 pandemic, whereas season and age were more relevant before the pandemic [12,13].

Surgical intervention emerged as a significant predictor of prolonged LOS in our study, with patients undergoing surgery nearly twice as likely to experience extended stays (AOR=1.966; p=0.024). This is consistent with Kashkooe et al. and may be due to the complexity of surgical care, rehabilitation needs, and postoperative monitoring [11]. In contrast, in ICU settings, Böhmer et al. identified factors such as massive transfusion, mechanical ventilation, and low Glasgow Coma Scale scores as major contributors to prolonged stays [14].

Interestingly, patients admitted due to RTAs were less likely to have prolonged LOS compared to other trauma mechanisms (AOR = 0.294; p = 0.009). This might be due to injury patterns, emergency response times, or streamlined management pathways. Similar findings have been reported in trauma populations with variability in hospitalization duration [11]. However, Alghnam et al. reported that RTAs were a major cause of prolonged LOS in pediatric patients [15]. Furthermore, Ngui et al. found a positive correlation between LOS and illness severity in Kenyan emergency department patients [16].

Limitations

The findings of this study are subject to several limitations. First, this was a single-center study, which may introduce selection bias, limit sample representativeness, and reduce generalizability. The long study period (2009-2014) may further limit the applicability of the findings to current trauma systems and surgical practices. Second, several key clinical confounders were not available in the medical records, including Injury Severity Score, Glasgow Coma Scale, patient comorbidities, ICU admission, in-hospital complications, discharge destination, and financial or insurance-related factors, which may have influenced LOS. Third, missing data on LOS may have affected the precision of the analysis. Finally, the retrospective design inherently limits data completeness, standardization, and causal inference.

## Conclusions

The prevalence of trauma patients who had prolonged LOS was common. Increasing age and surgical intervention were identified as the significant independent predictors of prolonged LOS. Interestingly, patients who had hospital admission due to RTA had a shorter duration of LOS. The findings of this study can guide healthcare providers and authorities in devising strategies to manage trauma patients and improve patient care. Rapid response, training of emergency service personnel, use of triage protocols, continuous training on advanced trauma life support, and monitoring for the early detection of complications are keys to improving health outcomes among trauma patients.
